# The complete mitochondrial genome of the scorpion *Centruroides vittatus* (Arachnida: Scorpiones)

**DOI:** 10.1080/23802359.2017.1407685

**Published:** 2017-11-25

**Authors:** Tsunemi Yamashita, Douglas Rhoads, Jeff Pummill

**Affiliations:** a Department of Biological Sciences, Arkansas Tech University, Russellville, AR, USA;; b Department of Biological Sciences, University of Arkansas, Fayetteville, AR, USA;; c Arkansas High Performance Computing Center, University of Arkansas, Fayetteville, AR, USA

**Keywords:** Mitogenome, scorpion, arachnid, Buthidae

## Abstract

The complete mitochondrial genome (mitogenome) of the Striped scorpion (*Centruroides vittatus*) was assembled from Illumina-based whole genome sequencing. The circular genome is 14,602 bp in length with 13 protein coding genes, 21 tRNA, two rRNAs, a translocation-inversion of tRNA^Leu^ compared to the horse shoe crab mitogenome, and the absence of tRNA^Asp^. The A + T content of the mitogenome is 68.1%. Our Bayesian and maximum likelihood phylogenetic analyses placed the *C. vittatus* mitogenome as a sister group of *C. limpidus* and nestled within the new world Buthids.

## Introduction

Arachnids are a diverse and ancient arthropod taxa, yet they are under represented in genomic studies, including mitochondrial genomes (165 arthropod species/2477 arthropod species). Within the arachnids, the bulk of mitogenomes submitted to NCBI (accessed September 2017) are of ticks and mites (107 species). Other arachnid mitochondrial genomes housed in NCBI include spiders (36 species), scorpions (eight species), harvestmen (three species), sun spiders (two species), whip spiders (two species), pseudoscorpions (two species), and whip scorpions (two species). The Buthid scorpion family compose the majority of the published scorpion mitogenomes; however, within the genus *Centruroides*, only *C. limpid*us is published. Here, we present the complete mitogenome for the striped scorpion, *C. vittatus*.

The striped scorpion, *C. vittatus*, is a common scorpion in the Midwestern United States and the northeastern states of Mexico. Although, it is medically important to humans, due to its neurotoxic venom, its venom appears to show reduced adverse effects compared to the western *C. sculpturatus*.

Total genomic DNA was extracted from a male scorpion collected in Pope County, AR (35° 31′36.96″N, 93° 08′29.21″W) with the Fast ID genomic DNA extraction kit (Genetic IDNA, Inc.). The genomic DNA quality was analysed through 0.9% agarose gel electrophoresis and by UV spectroscopy. The genomic DNA was sent to the DNA sequencing core facility at UAMS (Univ. AR Med. Sci.) for library generation and 2 × 300 paired end sequencing in a Illumina MiSeq small genome sequencer. The de novo assembly was conducted at the High Performance Computing Center at the University of AR-Fayetteville. The raw sequence data were processed (adapter removal, quality trimming, and merging of data) with BBmap (Bushnell 2016). The processed data were assembled with SPAdes v3.6.1 (Bankevich et al. 2012) and Ray v2.3.1 (Boisvert et al. 2010) with quality assessment with the program Quast v.4.0 (Gucrevich et al. 2013). The mitogenome was extracted from the assembly, confirmed by template reassembly using NGen (DNAStar 12.3) and annotated using BASys v1.0 (Van Domselaar et al. 2005) and with reference alignment to the published *C. limpidus* mitogenome (Dávila et al. 2005). The finished genome was deposited in NCBI with the accession number BankIt2047317 *Centruroides* MF975702.

The *C. vittatus* mitogenome matches the published *C. limpitus* mitogenome (81.6%) with a similar genome size (14,602–14,519 bp in *C. limpitus*) and order of the 13 protein coding genes, 21 tRNA, and two rRNAs. Both *Centruroides* mitogenomes differ from the horseshoe crab mitogenome with a translocation-inversion of tRNA^Leu^ and the absence of tRNA^Asp^. Both genomes are similar in size to other scorpion genomes, but smaller than for *Mesobuthus martensii* (15,034 bp) (Choi et al. 2007). The A + T content of the *C. vitttatus* mtDNA assembly is also similar to *C. limpidus* (68.1–64.46%, respectively). Most of the nucleotide variation between the *C. vittatus* and *C. limpidus* mtDNA genome occurs in the putative non-coding control region with *C. vittatus* showing a 97 bp insertion.

**Figure 1.**
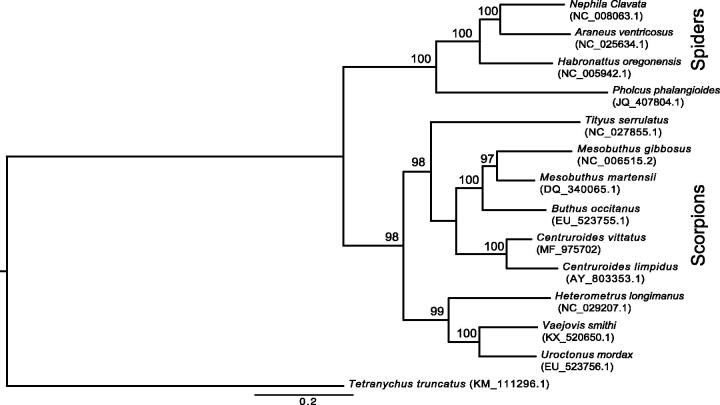
A 50% majority-rule consensus tree created in Mr. Bayes for the scorpion, spider, and mite mitogenomes. The Bayesian clade credibility values are shown in the tree with the number of substitutions per site shown in the scale.

We created a phylogeny showing the placement of the *C. vittatus* mitogenome with respect to eight scorpion, four spider, and one mite mitogenomes. The consensus tree was created through Bayesian methods with MrBayes v3.1.2 (Ronquist and Huelsenbeck 2003). The mite *Tetranychus truncatus* was selected as the outgroup (Figure 1). A phylogenetic second tree was created with maximum likelihood methods in RAxML v8.0.0 (Stamatakis 2014). As this tree was identical to the Bayesian tree, we only present the Bayesian tree.
